# A brief history of otologic surgery: restoring hearing through the ages

**DOI:** 10.1007/s00405-026-10049-7

**Published:** 2026-03-05

**Authors:** Christian Alessio, David Sunnucks

**Affiliations:** 1 Faculty of Medicine, Barts and the London School of Medicine and Dentistry, Queen Mary University of London, Malta Campus, Victoria, Malta; 2 Department of Anatomy, Barts and the London School of Medicine and Dentistry, Queen Mary University of London, Malta Campus, Victoria, Malta

**Keywords:** Otologic surgery, History of otology, Developments in ear surgery, Myringotomy, Mastoidectomy, Stapedectomy, Myringoplasty

## Abstract

**Background:**

With an aging demographic, hearing loss is set to become the most common sensory deficit worldwide. Hearing impairment has a profound and far-reaching impact on wellbeing, extending well beyond the immediate challenges of sound perception.

**Objective:**

This review aims to trace the evolution of surgical practices in otology from ancient to modern times, emphasizing key milestones that have shaped the field.

**Methods:**

A narrative historical review was conducted examining major developments in otologic surgery, with particular focus on contributions from key figures including Hippocrates, Riolan, Kessel, Shea, Berthold and House. The review centers on the management of conductive hearing pathologies, specifically ear infections, ossicular and bony abnormalities, and tympanic membrane perforations.

**Results:**

Historical analysis demonstrates a progressive refinement of surgical techniques, driven by anatomical discoveries, technological advancements, and innovative operative approaches. Contributions from pioneering surgeons significantly advanced the management of conductive hearing disorders and laid the foundation for modern otologic surgery.

**Conclusion:**

The evolution of otologic surgery reflects the transformative impact of medical, surgical and technological innovation on hearing restoration. Understanding this historical progression provides essential context for current practice and highlights potential avenues for future advancement.

## Introduction & background

### Epidemiology

**Fig. 1:**
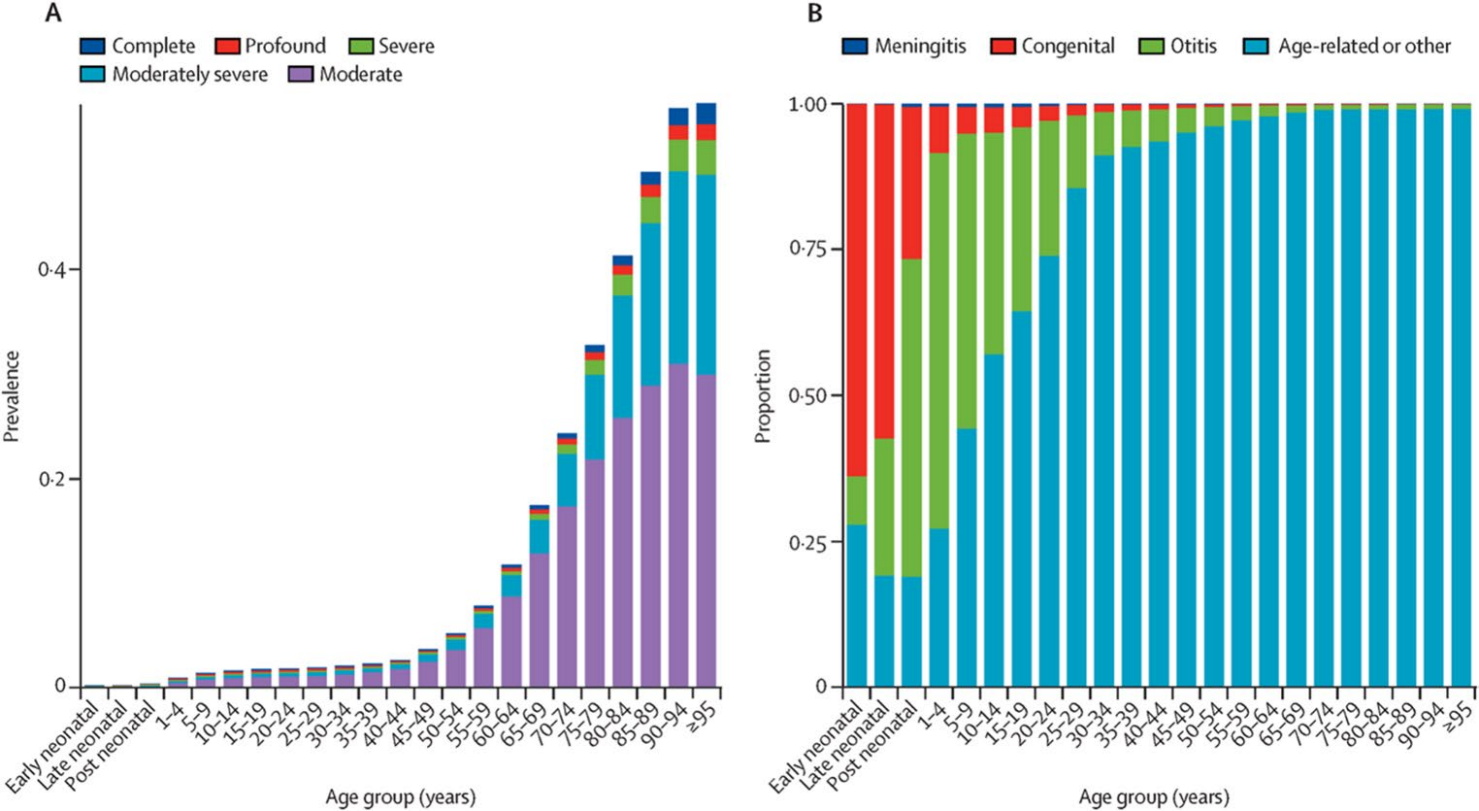
Prevalence and aetiology of hearing loss across age groups. Prevalence of hearing loss (>35 dB) by age and severity (**A**) and proportion of hearing loss by age and cause for all severities (**B**). Prevalence of hearing loss increases with increasing age. Congenital causes are most common in the neonatal period, otitis is the most common cause in childhood and age-related/other most common in adulthood. *Reproduced with permission from Haile et al., The Lancet (2021) 397:10278, licensed under CC BY 3.0*

According to the latest estimates from the Global Burden of Disease (GBD) Study 2019, over 1.5 billion people worldwide suffered some degree of hearing impairment, with prevalence rising sharply with age. Older adults are disproportionately affected by hearing problems compared to the general population, and hearing loss constitutes a leading cause of disability in later life Fig. 1 [1]. In fact, hearing impairment carries a substantial medical, psychological and socioeconomic burden with well-established associations with social isolation, unemployment, depression, cognitive decline and frailty [2].

HL is defined as a partial or complete inability to hear sounds in one or both ears. It can be described by its course, severity, and type. HL may be sudden or progressive, stable, or fluctuating. Anatomically, it can be classified as conductive (CHL), sensorineural (SHL), or mixed (MHL). CHL is characterized by impaired transmission and transduction of sound waves to mechanical vibrations and is associated with outer and/or middle ear pathology, that is anywhere from the pinna to the footplate of the stapes. Conversely, SHL is characterized by impaired transduction of pressure waves into neuroelectric signals and occurs as a result of damage to the cochlea, auditory nerve, and/or higher auditory centers. MHL, as the name implies, is caused by a combination of conductive and sensorineural deficits.

## Methodology

### Objectives

This review aims to provide a brief historical account of otologic surgery, with a particular focus on the impact of advances in medical knowledge, surgical techniques, and instrumentation on the management of hearing loss over the years. To achieve this, this paper traces the evolution of otology from its inception to present times, highlighting some of the key milestones and technological innovations that have influenced current practices. Given a prominent historical focus on the surgical management of conductive hearing pathologies, three main types of auditory disorders are discussed: otitis media, otosclerosis, and tympanic membrane perforation.

### Search strategy

A comprehensive literature search was conducted to explore the major historical developments in otologic surgery. The databases used included PubMed, JSTOR, and ScienceDirect. The search terms were a combination of keywords such as ‘history of otorhinolaryngology’, ‘developments in ear surgery’, ‘milestones in otologic surgery’, and ‘evolution of surgical techniques in otology’. The search focused primarily on systematic reviews and original peer-reviewed research articles between 1970 and 2024. The former were used to explore the historical context, while the latter served to detail the progression of surgical techniques over the years.

### Limitations

Although efforts were made to gain a global perspective, research was limited to English-based sources that mainly reflect European and North American practices.

## Key milestones in otologic surgery

### Historical perspective: anatomical insights and surgical breakthroughs

The birth of otology dates back thousands of years, with the earliest recorded descriptions of ear disease found in Egyptian, Greek, and Hindu texts. The Egyptians were one of the first civilizations to write about hearing disorders. In the Ebers papyrus (4000 BCE), they referred to otitis media as ‘fire in the heart of the ear’ and recommended honey as a treatment. Not until three thousand years later did the pre-Socratic philosopher Empedocles (490 − 430 BCE) provide a crude anatomical description of the cochlea (from the Greek *kókhlos*), likening its spiral contours to those of a seashell found in the Mediterranean region. Around 400 BCE, Hippocrates described the tympanic membrane and mastoid air cells, identifying acute and chronic middle ear infection as a potential cause of deafness. By 300 BCE, Plato’s greatest disciple, Aristotle, theorized that the inner ear acted as a resonating chamber that vibrated in response to sound, producing the very first theory of hearing [3].

In *De Medicina*, Aulus Cornelius Celsus recommended the use of a probe and an incision with a primitive scalpel for partial and complete aural atresia, respectively. He also established the importance of ear irrigation in the treatment of cerumen impaction, using a mixture of vinegar and soda to soften the wax and then flushing the external auditory canal with a combination of vinegar, wine, and honey. With his work, Celsus is credited with one of the earliest descriptions of otologic surgery. For the next 15 centuries, Galen’s teachings dominated medical thought, shaping Western medicine [4]. In addition to his prominent advocacy for Hippocrates’ humoral doctrine, the Greek physician made pioneering discoveries in human anatomy and physiology. He drew a distinction between sensory and motor nerves and theorized that sound waves were transmitted to the brain via the auditory nerve [5]. Additionally, Galen proposed various medical and surgical interventions to treat ear-related ailments, including drainage and foreign body removal techniques [4].

However, it was not until the Renaissance that the field of otology saw significant progress in terms of both anatomical understanding and surgical techniques. In fact, if knowledge was limited before to the study of animals and the treatment of wounded gladiators, this changed drastically as humanism spread across Europe, shifting the focus from religious dogma to rational enquiry. Through human dissection, anatomists carefully unraveled the hidden complexities of the human ear. In 1543, Andreas Vesalius provided detailed descriptions of the middle ear, including the malleus, incus, and the oval and round windows. Three years later, Giovanni Filippo Ingrassia described the stapes, completing the anatomical understanding of the ossicular chain [5].

Later in the 16th century, Bartolomeo Eustachi provided detailed anatomical descriptions of the Eustachian tube, semicircular canals and cochlea [3]. Drawing on Cardano’s bone conduction experiments, Hieronymus Capivacci differentiated conductive from sensorineural hearing disorders [6]. In 1649, Riolan the Younger revisited Hippocratic teachings on the management of otitis media with effusion, advocating fluid drainage to ‘balance the humours’ and restore hearing. He intentionally incised the tympanic membrane with the use of an ear spoon, performing the first formal myringotomy [7]. At the end of the 17th century, Joseph Du Verney published the first formal text on otology, *Traité de l’organe de l’ouïe*, a detailed illustrative account on inner ear anatomy [6].

**Fig. 2:**
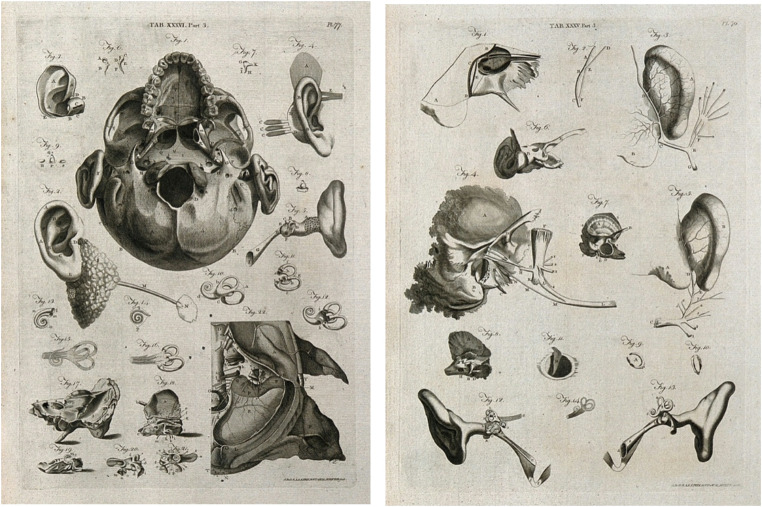
Historical depictions of the human ear and adjacent structures. Line engravings by A. Bell after the works of Du Verney and Valsalva, originally published in 1798. These plates depict detailed illustrations of external, middle and inner ear structures, capturing the state of anatomical knowledge at the end of the 18th century. *Original public domain images from the Wellcome Collection*

In the early 1700s, after extensive dissection of more than 1,000 human specimens, Antonio Maria Valsalva further elucidated the physiological configuration of the ear, substantiating the work of former anatomists Fig. 2. In particular, he was the first to define the three anatomical divisions of the ear: outer, middle and inner [8]. He also documented cases of stapes fixation, providing one of the earliest recorded descriptions of otosclerosis [9].

The nineteenth century ushered in new insights and techniques that laid the foundation for modern otology. Domenico Cotugno and Antonio Scarpa identified the presence of fluid –perilymph and endolymph – in the inner ear, challenging a theory that persisted for centuries and forming the basis for the understanding of hearing physiology [3]. Following in the footsteps of Jean Riolan the Younger, Sir Astley Cooper presented two papers to the Royal Society in 1801 in which he established the clinical indications for myringotomy and provided a detailed outline of the steps of the procedure. Cooper proposed that surgery be reserved solely for cases of conductive hearing loss secondary to Eustachian tube obstruction and recommended perforation of the anteroinferior quadrant to preserve the integrity of the ossicular chain [7].

Around the mid-1800s, Alfonso Corti described the microscopic structures of the cochlea with the aid of a compound microscope, providing a detailed account of the sensory epithelium, eponymously named after him [10]. Later, Joseph Toynbee published a comprehensive catalogue compiling pathological analysis of more than 1,500 human ears. Through extensive dissection, Toynbee demonstrated that the main cause of hearing impairment was secondary to inflammatory processes in the middle and inner ear rather than the auditory nerve. He also invented the otoscope and proposed treatments for common ear disorders [3, 4, 11]. By the end of the nineteenth century, Adam Politzer gave a rudimentary definition of otitis media with effusion, noting its association with antecedent upper respiratory tract infections (URTI), and devised a minimally invasive technique to equalize middle ear pressures. This involved the insufflation of air into the Eustachian tube by means of a rubber bag, now commonly referred to as the Politzer bag. Though revolutionary for its time, Politzerization has now been largely superseded by more effective procedures. The diligent work of Kessel, Berthold, Wilde, Wüllstein, Zöllner, and Jansen further advanced the field of otologic surgery, providing the earliest recorded descriptions of stapes mobilization, myringoplasty, tympanoplasty and labyrinthectomy [3, 4].

In particular, the late nineteenth and early twentieth centuries marked a period of pivotal advancements that profoundly redefined the field of surgery, a discipline that may have otherwise stagnated and eventually been abandoned. In fact, many procedures that were once considered standard practice would now be viewed not only as unethical and inhumane, but also excessively dangerous, given the substantial risk of serious and life-threatening complications. Figures of the likes of Morton, Lister, and Fleming revolutionized surgical practice. In 1846, William Thomas Green Morton demonstrated ether’s anaesthetic superiority over nitric oxide during the surgical excision of a neck tumour [12]. Twenty years later, influenced by Pasteur’s germ theory, Lister laid the foundation for the use of the antiseptic technique (“no germs, no infection, no disease”), advising surgeons to sterilize their hands and instruments before and after every procedure with 5% carbolic acid solution [13]. In 1928, Alexander Fleming determined the antibacterial properties of penicillin, marking the start of the antibiotic era [14]. These were seminal discoveries that dramatically altered the course of surgery.

### Modernizing otologic care: diagnostic and surgical innovations

With the isolation and extraction of the first antimicrobial agents, the focus of medicine began to shift toward prevention rather than cure, the need for radical surgeries waned, and more importance was given to hearing preservation [3, 4]. This was encouraged by advancements in diagnostics, rehabilitative devices, and surgical instrumentation.

In 1898, drawing on Edison, Berliner, and Bell’s pioneering discoveries, Hutchison devised the first electronic hearing aid, called the Akouphone. This consisted of a microphone, amplifier, headphones, and battery and used a portable carbon transmitter to convert weak signals into louder, audible sounds. This marked a pivotal moment in history, representing a dramatic shift in both technology and design compared to earlier devices such as the acoustic chair and the ear trumpet. That being said, the Akouphone only increased decibel levels by about 15 dB; this combined with its cumbersome design and steep retail price made the device ineffective, impractical, and inaccessible. It was the advent of vacuum tube technology and later transistor that led to improved sound amplification and more compact hearing devices [15].

Up until the end of the nineteenth century, Weber and Rinne’s hearing tests were among the most accurate and systematic methods to assess audiologic function and differentiate between CHL and SHL. The former is carried out by holding a vibrating tuning fork (512 Hz) anywhere in the midline of the skull to assess lateralization of sound, whereas the latter is conducted by placing a struck tuning fork alternately on the mastoid bone and next to the external acoustic meatus to evaluate whether bone conduction is greater than air conduction [16]. It was only in 1923 that, inspired by the work of Carl Seashore, Fletcher, Fowler, and Wegel developed the first standardized audiometric examination. This involved testing hearing thresholds by means of a rudimentary audiometer (frequency range: 32 − 16,384 Hz), specially designed headphones or loudspeakers, and a bone conduction vibrator. The hearing sensitivity would then be plotted against the sound frequency on an audiogram to facilitate interpretation [17]. In contrast to early hearing tests, Fletcher, Fowler, and Wegel’s audiometric exam was the first quantitative method of hearing assessment that provided a more systematic and objective measurement of hearing loss, laying the foundation for modern audiology.

In 1921, Nylen recognized the need for increased magnification in otologic surgery and introduced the monocular operating microscope. This was rapidly replaced by a binocular model developed by the Zeiss Optical Company one year later. Collectively, these discoveries greatly improved surgical precision and contributed to better clinical outcomes [18]. The introduction of fibreoptic endotoscopes to examine the middle ear in the 1960s further advanced the field, paving the way for the development of minimally invasive ear surgery [3].

A summary of key historical milestones can be found in Table 1.

**Table 1:** Summary of the key milestones in otology

Year	Milestone	Proponent
440 BC	Crude description of the cochlea	Empedocles
400 BC	Description of tympanic membrane and mastoid air cells; identification of middle ear infection as a common cause of deafness	Hippocrates
14–37 CE	Ear irrigation for cerumen impaction; use of probe/scalpel for congenital aural atresia	Celsus
200 CE	Identification of sensory and motor nerves; description of auditory nerve as a conduit for hearing	Galen
1543	Description of malleus, incus and oval and round windows	Vesalius
1546	Description of stapes	Ingrassia
1561	Description of Fallopian canal, tympanic membrane, vestibule and cochlea	Fallopius
1570	Description of Eustachian tube, semicircular canals and cochlea	Eustachi
1603	Introduction of bone conduction testing; distinction between conductive and sensorineural hearing loss	Capivacci
1649	First myringotomy for otitis media with effusion	Riolan
1683	First formal text of otology (*Traité de l’organe de l’ouie*)	Du Verney
1700	Second formal text of otology (*De Aure Humana Tractatus*); first documented cases of otosclerosis; first account of mastoid surgery	Valsalva, Petit
1711	**Invention of the tuning fork**	**Shore**
1800	Description of perilymph and endolymph	Cotugno, Scarpa
1801	Definition of indications for myringotomy	Cooper
1834	Introduction of Weber’s test	Weber
1838	Description of cholesteatoma	Müller
1846	**Discovery of anaesthesia**	**Morton**
1851	Description of cochlear structures (Organ of Corti); invention of the otoscope	Corti, Toynbee
1855	Introduction of Rinne’s test	Rinne
1857	Postaural incision for mastoid exploration	Wilde
1861	Description of triad of Ménière disease: periodic vertigo, hearing loss and tinnitus	Ménière
1865	Politzerization and myringotomy for treatment of serous otitis media	Politzer
1866	**Introduction of the aseptic technique in surgery**	**Lister**
1876	Invention of the telephone	Bell
1878	First stapes surgery for otosclerosis (stapes mobilization); myringoplasty using skin flap from the forearm	Kessel, Berthold
1883	Description of pathophysiology of otosclerosis	Politzer
1898	**Invention of the first electric hearing aid (Akouphone)**	**Hutchison**
1921	Introduction of the monocular operating microscope	Nylen
1922	**Introduction of binocular operating microscope**	**Zeiss Optical Company**
1923	First standardized audiometric examination	Fletcher, Fowler and Wegel
1928	**Discovery of penicillin**	**Fleming**
1938	One-stage fenestration procedure for otosclerosis	Lempert
1940	Electrical burr for mastoid surgery	Boettcher
1950	First successful tympanoplasty	Wullstein and Zöllner
1955	Revision of stapes mobilization	Rosen
1956	First successful stapedectomy and reconstruction	Shea
1961	First successful cochlear implantation	House

Highlighted in bold are landmark innovations that significantly advanced clinical diagnostics and surgical practice. The rest are minor, supporting innovations whose incremental contributions complemented major developments [3, 4].

## Surgical advances in conductive hearing loss

The following paragraphs focus on three of the most common historical causes of CHL, namely otitis media, otosclerosis, and tympanic membrane perforation. By exploring the aetiology and management, this section aims to provide a brief overview of the underlying pathophysiology and trace the evolution of surgical interventions, placing particular emphasis on evaluating the effectiveness of different techniques.

### Otitis media

Otitis media (OM) refers to a spectrum of inflammatory disorders of the middle ear characterized by ear discharge or effusion. Depending on its duration, frequency and associated clinical and otoscopic findings, it is classified into three main types: acute OM (AOM), OM with effusion (OME), and chronic suppurative OM (CSOM) [19].

AOM is an acute, suppurative infection of the middle ear, almost always preceded by a URTI. It is generally bacterial in origin; however, viral coinfection may also occur. AOM begins with viral-induced inflammation of the nasopharynx and Eustachian tube. Tubal dysfunction follows, leading to negative middle ear pressure and bacterial/viral migration and colonization of the middle ear cavity. This triggers the activation of both innate and adaptive immune responses, resulting in suppurative inflammation and potentially tissue damage. Clinically, AOM is characterized by rapid onset of otalgia, typically unilateral, and nonspecific symptoms of systemic upset, including fever, irritability, and vomiting. The diagnosis is confirmed by the presence of a bulging and erythematous or opacified tympanic membrane on otoscopic examination and decreased mobility of the tympanic membrane on pneumatic otoscopy [19].

OME is defined as a persistent middle ear effusion in the absence of signs of acute infection. In contrast to AOM, the effusion is serous or seromucinous in nature and generally lasts three months or longer. The pathogenesis is complex and poorly understood but is believed to involve a combination of chronic low-grade chronic inflammation and Eustachian tube dysfunction that leads to mucosal hyperplasia and goblet cell proliferation. The most common symptoms are hearing loss and a sense of aural fullness; thus, clinicians should maintain a high index of suspicion in children presenting with persisting hearing difficulties, speech or language delay, and behavioural or sleep disorders. On examination, an air-fluid level is visible and the tympanic membrane is opalescent rather than opaque and mobility is reduced [20].

CSOM is a chronic, suppurative infection of the middle ear and mastoid cavity, commonly resulting from partially treated or untreated AOM. Malignant CSOM is a more aggressive form associated with severe complications, including cholesteatoma, tympanosclerosis, osteitis, labyrinthitis, and intracranial abscesses. The pathogenesis of CSOM is multifactorial and, like OME, not fully elucidated, although it is believed to involve a cycle of inflammation, ulceration, infection, and scarring that eventually leads to bone erosion and destruction. Clinically, patients often present with a two-week history of persistent, purulent otorrhea, and unilateral hearing impairment. The diagnosis is confirmed by direct visualization of the perforation on otoscopy [21].

#### Evolution of surgical approaches

While the mainstay of treatment for uncomplicated OM is antibiotic therapy, especially when bacterial aetiology is suspected and spontaneous resolution is unlikely, surgery is reserved for recurrent, chronic, or complicated cases where medical management alone is inadequate. The choice of surgical intervention varies depending on each case, but generally centers around myringotomy with/without tympanostomy, tympanoplasty, and mastoidectomy. Over the years, the surgical management of OM has evolved dramatically, providing enhanced precision, minimal invasiveness, and ultimately better patient outcomes.

Myringotomy, also known as tympanotomy, involves making a small radial incision in the anteroinferior quadrant of the tympanic membrane to drain effusions from the middle ear. Generally, this is followed by placement of a tympanostomy tube or grommet, a temporary device varying in design, material, and composition that allows for ventilation of the middle ear and pressure equalization. This procedure is recommended in cases of recurrent AOM (*≥* 3 episodes in the previous 6 months) and OME, to break the cycle of inflammation and restore hearing [22]. The practice of myringotomy dates back to the mid-1600s; however, while the basic principles remain unchanged, the technique and instrumentation have advanced considerably.

Historically, surgery was performed blindly by means of primitive scalpels (e.g. ear spoon, sickle knife), often resulting in crude incisions that would either close rapidly or cause excessive damage to surrounding structures [7]. Current techniques instead employ an endoscopic approach and fine microsurgical instruments (e.g. otoendoscope, myringotomy blade, Frazier suction tip, and alligator forceps) that allow for direct visualization of the tympanic membrane, greater cutting precision, refined tube insertion and reduced trauma [23, 24]. Laser-assisted procedures have also been developed, though less widely adopted in clinical practice. These involve the creation of an opening in the tympanic membrane by means of a carbon dioxide laser system (ESC Sharplan, Yokneam, Israel), whose power is adjusted according to the thickness of the membrane. Laser-assisted tympanic membrane fenestration (LTMF) provides longer lasting patency compared to cold-knife myringotomy (2–4 weeks vs. 48–72 h) and is therefore the preferred surgical approach in cases where medical management has failed but tympanostomy is not recommended [25, 26].

Since their earliest use, grommets have undergone significant refinements in material selection, design, and overall functionality. In an attempt to reduce recurrence, oiled catgut strings, lead wires, whale bones, and wedge-shaped excisions were initially trialed to prevent spontaneous membrane closure, but to no avail. It was Politzer who pioneered the development of modern tympanostomy tubes with the introduction of the rubber grommet [7]. Over the past decades, significant efforts have been made to identify more biocompatible materials and optimize tube design with the aim of improving longevity, reducing failure rates, and minimizing complications. Today, fluoroplastics and silicones are preferred over metals (e.g. stainless steel, gold, and titanium) given their comparatively stronger heat resistance and chemical inertness [22]. However, the available evidence is contradictory and no single study has been able to prove a statistically significant advantage in using one material over the other. This could be due to individual patient factors (e.g. age and Eustachian tube function), specific medical considerations (e.g. insertion and retention time), and tube manufacture (e.g. inherent and surface composition, structure and size). Ultimately, tube selection should be tailored to the patient’s needs and clinical picture after a detailed expert evaluation [27].

Mastoidectomy involves the erosion of infected or diseased segments of the mastoid cortex and subjacent air cells. This is generally recommended for the definitive treatment of chronic OM and cholesteatoma. The procedure is performed by making an incision posterior to the postauricular sulcus to expose the mastoid cortex and allow direct visualization of the affected tissues. Endoaural incisions can sometimes be performed, extending from the tragus to the root of the helix along the posterior canal wall; however, their use is limited as they provide a comparatively limited surgical field to postaural incisions^46^. Depending on the extent of the procedure, mastoidectomy is broadly classified into two main types: canal wall up (CWU) and canal wall down (CWD) [28].

CWU involves removal of diseased tissue with preservation of the posterosuperior wall of the external osseous auditory canal. Depending on anatomical boundaries, CWU procedures are further categorized into simple and complete. In simple mastoidectomies, the drilling does not extend beyond the Körner septum, whereas in complete mastoidectomies, the mastoid cortex, antrum, and all lateral air cells are removed [28].

CWD involves dissection of both healthy and diseased tissue, including resection of the posterior wall. Similar to CWU mastoidectomies, CWD operations may be performed using a radical or modified radical approach. In radical mastoidectomies, complete exenteration of middle ear structures is performed (the stapes is usually left in situ), while in modified radical mastoidectomies, the ossicular chain and tympanic membrane are preserved. A conservative approach is typically preferred for localized pathology (e.g. acute mastoiditis and subperiosteal abscess) to achieve definitive resolution. In contrast, radical procedures are reserved for cases of extensive disease (e.g. chronic OM and recurrent cholesteatoma) to mitigate the risk of intracranial extension and recurrence [28].

**Fig. 3:**
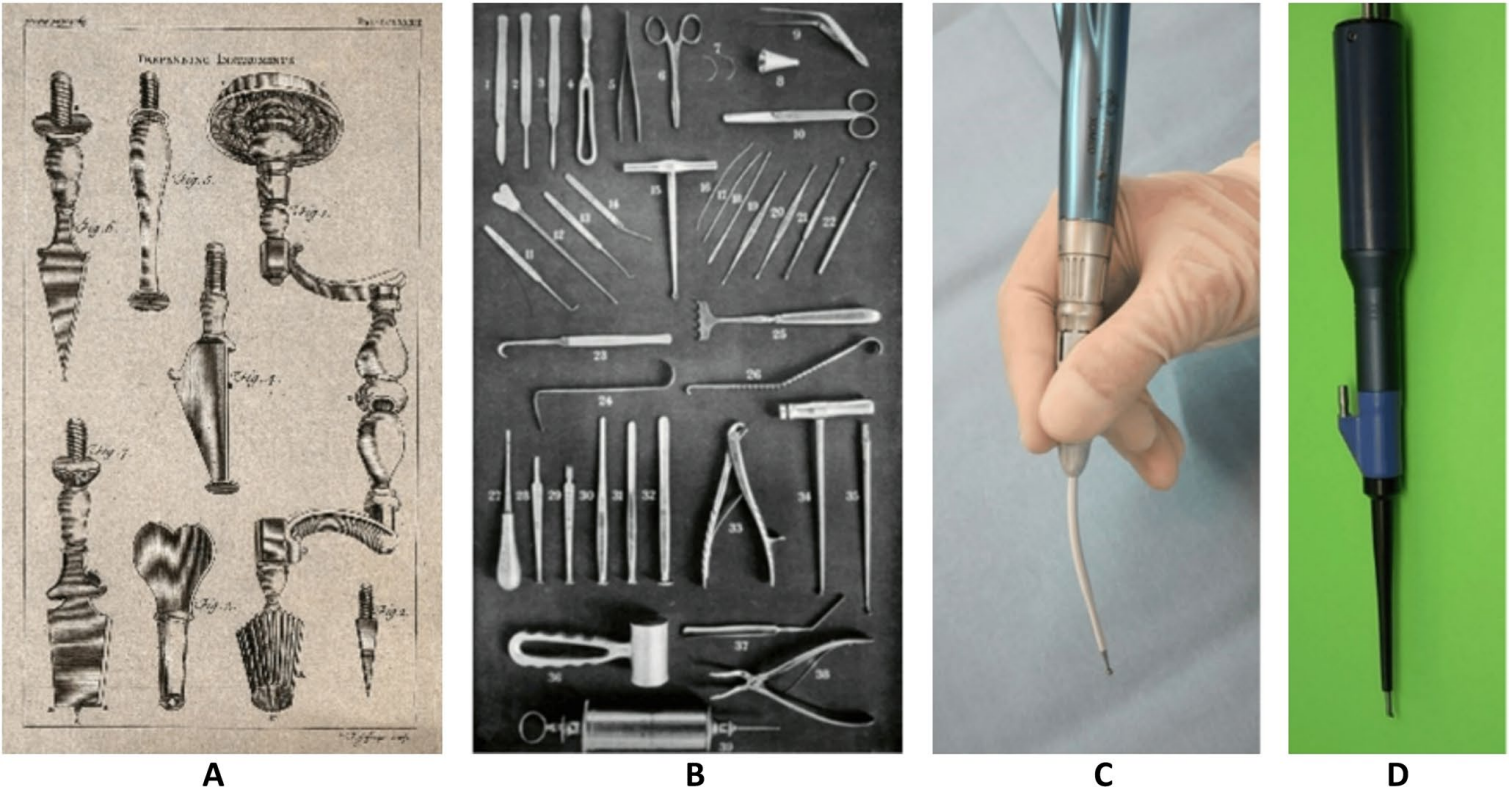
Evolution of surgical instrumentation for mastoidectomy. From rudimentary trepans (**A**) to highly precise tools (**B**) including the Visao® high-speed otologic drill (**C**) and the Sonopet® Omni ultrasinic surgical aspirator (**D**). *Adapted from Ito et al., Applied Sciences (2019) 9:4, licensed under CC BY 3.0*

Historically, mastoidectomy was almost always an ablative procedure, involving indiscriminate removal of bone and soft tissue and often resulting in permanent hearing loss [29]. In the late 19th and early 20th centuries, surgery was performed blindly by means of trepans, chisels, gouges, and hammers and little care was given to balancing disease eradication with functional preservation Fig. 3. The introduction of the electric drill and later on high-speed drills with diamond and cutting burrs revolutionized surgical practice. These discoveries have significantly improved precision, minimized complications, and cut down operative time [30]. In addition, the development of advanced imaging modalities such as computed tomography (CT), along with facial nerve monitoring (FNM), has considerably improved both preoperative planning and intraoperative decision making. In a prospective study examining FNM data from 52 otologic procedures (28 tympanomastoidectomies), FNM has been shown to reduce the risk of nerve damage in 100% of high-risk cases (39.2% due to pathologic changes, 7.8% due to anatomical variants) and reduce operative time in 86.5% of procedures [31].

### Otosclerosis

Otosclerosis (OS) is a progressive osseous dysplasia of the otic capsule characterized by pathological bone remodeling and subsequent ankylosis. Depending on the site of anatomical involvement, it is classified into two main types: fenestral or stapedial and retrofenestral or cochlear. Fenestral OS affects the oval window and the footplate of the stapes and leads to CHL due to ankylosis of the stapediovestibular joint. In advanced disease, the involvement of the bony labyrinth may produce an MHL pattern. In contrast, retrofenestral OS affects the cochlea and leads to SHL due to hyalinization of the spiral ligament and atrophy of the stria vascularis [32]. Stapedial pathology accounts for the vast majority of cases, and isolated cochlear involvement is rarely encountered in clinical practice. In fact, the presence of localized cochlear disease should prompt further evaluation to exclude other primary bone disorders such as osteogenesis imperfecta and Paget’s disease of the bone [33].

Although key pathophysiological mechanisms have been established, the exact pathogenesis remains unclear. Patients most commonly present with a longstanding history of bilateral hearing loss that has gradually worsened over many years. The diagnosis is mainly clinical and is based on otoscopic (e.g. normal tympanic membrane and no signs of effusion or infection in the middle ear) and audiometric findings (e.g. CHL and Carhart notch) [34].

#### Evolution of surgical approaches

Stapes surgery is the workhorse of OS management. Two main procedures are currently used to correct or bypass the ossification and fixation of the stapes bone and restore sound conduction through the ossicular chain: stapedectomy (older) and stapedotomy (newer). Both techniques involve excision of the head, neck, and anterior and posterior crurae of the stapes, the key distinction being the preservation of the stapedial footplate [35].

Stapedectomy is based on a relatively older and more radical approach that involves total removal (the entire footplate removed) or subtotal removal (only portion of the footplate removed, usually the posterior or central) of the stapes and replacement by a prosthesis. In contrast, stapedotomy relies on a more modern and conservative technique that involves the creation of a small fenestra in the stapedial footplate, using a microdrill or laser, and then placement of a prosthesis [35, 36].

Despite the general preference for stapedotomy, given the comparable short- and long-term hearing outcomes and the supposedly lower risk of complications, existing research is conflicting [37, 38]. For example, in a retrospective study analyzing audiometric data from 145 patients who underwent primary stapedectomy (134) or small fenestra stapedotomy (75), no statistically significant differences were found in the early/late postoperative pure tone average (PTA), air-bone gap closure, speech discrimination scores, or incidence of SHL. Interestingly, the same review compared 42 patients who had both procedures performed (stapedectomy in one ear and stapedotomy in the other) and obtained similar results [39]. Other studies have shown better long-term audiometric results, especially in the high-frequency range (1–4 kHz), and lower rates of SHL with stapedotomy [40–42]. Ultimately, many variables influence clinical outcomes in stapes surgery, including size (0.3–0.8 mm) and material composition of the prosthesis (fluoroplastic vs. metal piston) but surgical expertise is believed to be the main contributor to surgical success [37, 38].

Stapes surgery has undergone considerable advances over the years. These can be broadly classified into five main historical periods: the early mobilization era, the fenestration era, the late mobilization era, and the stapedectomy/stapedotomy era. The very first attempt to treat stapes fixation was pioneered by Kessel in 1878. This involved excision of the sclerotic bone to loosen the stapediovestibular joint and restore its oscillatory range. Though revolutionary for its time, the Kessel mobilization technique was quickly abandoned due to the high incidence of infection and refixation. Holmgren then introduced the three-stage fenestration technique to bypass the otosclerotic focus altogether and provide an alternative pathway for sound conduction. This was achieved through the creation of a fenestra either in the promontory or in the bony labyrinth. Holmgren’s work was further refined by Lempert, who instead proposed a single-stage fenestration technique. Again, these procedures provided only temporary hearing improvement as the perforation would rapidly heal; not to mention the significant risk of postoperative complications such as SHL due to damage to the vestibular apparatus. In 1953, Rosen refined Kessel’s procedure by rejecting his radical approach and instead advocating gentle manipulation of the stapes, but to no avail. It was Shea who pioneered the use of a Teflon prosthesis to replace the ankylosed ossicle, paving the way for modern stapedectomies and stapedotomies [43].

It should be noted that, given their conservative approach, modified mobilization techniques have gained increasing interest in recent years. In fact, studies indicate that the outcomes are comparable to traditional procedures. A retrospective study that analyzed audiological data from 67 patients who underwent stapedectomy (32) and stapes mobilization (35), found no statistically significant differences in air-bone gap closure between the two groups [44]. Similarly, Shiao et al. reported a postoperative air-bone gap of < 20 dB in 102 of 106 patients (96.2%) who underwent minimally traumatic stapes surgery (MTSS) [45].

### Tympanic membrane perforation

Tympanic membrane perforation (TMP) refers to a tear in the tympanic membrane. This establishes a direct passage between the external auditory canal and the middle ear cavity, increasing susceptibility to infection. According to the site of anatomical involvement, perforations are broadly classified as central, marginal or attic [46]. The diagnosis of TMP is clinical, by direct visualization of the perforation on otoscopy. CT may sometimes be used to exclude damage to the ossicular chain and guide preoperative planning.

#### Evolution of surgical approaches

Most acute perforations heal spontaneously, whether the cause is infection or trauma. Thus, the mainstay of therapy for asymptomatic perforations is general precautions along with topical antibiotics, in the event of infection or contamination. Surgery, known as myringoplasty or tympanoplasty, is typically reserved for recurrent or high-risk cases. These include symptomatic central perforations, chronic perforations where conservative management alone has failed, and unsafe perforations, such as attic or marginal, where there is a substantial risk of damage to the structures of the middle ear, given their anatomical proximity [47].

Several techniques have been developed for TMP, with the underlay and overlay approaches being the two most frequently used in surgical practice. The former is generally recommended for small posterior perforations and involves placement of the graft posterior to the defect, medial to the annulus. The latter is typically reserved for anterior and total perforations and the graft is positioned anterior to the tympanic membrane remnant, lateral to the annulus [48]. Classically, grafts are harvested from the temporalis fascia, perichondrium or tragal cartilage [49]. Each technique has its advantages and disadvantages, and ultimately it is up to the surgeon to select the most suitable approach. While the underlay method provides simpler and faster repair, limited visibility and poor graft vascularity may lead to adhesions and graft failure. On the contrary, the overlay method provides a better healing rate but is more technically challenging, with a higher risk of blunting and lateralization of the graft [48].

Berthold was the first to attempt myringoplasty in the late 1800s using a full-thickness skin graft from the forearm. However, this approach was associated with poor postoperative outcomes due to high risk of rejection and infection of the graft and was eventually abandoned. In 1950, Wullstein and Zöllner further refined the technique by advocating the use of the temporalis fascia rather than full-thickness skin grafts. This new approach substantially improved graft integration and vascularization, dramatically reducing failure rates. In addition, they introduced a classification system for tympanoplasty, based on the extent of involvement of the middle ear. In general, Wullstein and Zöllner made groundbreaking advances that continue to serve as the cornerstone of modern tympanoplasty [50].

## Discussion

The operative management of conductive hearing pathologies has evolved dramatically over time. Historical challenges, including limited surgical access, visualization, and microsurgical precision, have underscored the importance of continual technological advancement and a persistent need for innovation.

Endoscopic ear surgery has emerged as a pivotal development in otologic surgery, improving exposure and reducing operative risk, and transforming procedures that were once lengthy and uncertain into minimally invasive, day-case operations with favourable outcomes. However, their adoption remains limited by access to specialised equipment, surgeon expertise and institutional resources, which may vary across regions and countries.

Looking ahead, robotic-assisted systems promise elimination of physiological tremor, enhanced microsurgical accuracy, improved access to confined anatomical spaces, and shorter operative times. Yet, their clinical efficacy and cost-effectiveness in otology remain to be fully established. Similarly, artificial intelligence (AI) and machine learning models have the potential to streamline diagnostic pathways, reduce misdiagnosis, prevent unnecessary surgeries, and optimize preoperative planning; however, their safe implementation into clinical practice depends on high-quality data collection and robust validation.

The integration of surgical, robotic, and computational tools holds tremendous potential in otology but current limitations emphasise the need for continued research, training and collaborative efforts among all stakeholders to ultimately ensure the provision of safe, effective, and equitable care.

## Conclusion

The history of otologic surgery is one of relentless innovation and progress, driven by a growing understanding of ear anatomy, pathology, and technological advancements. The key developments highlighted in this review are a testament to human ingenuity and the continual pursuit for surgical precision and success.

The evolution of otologic surgery clearly demonstrates how techniques have progressed from rudimentary, experimental procedures to sophisticated, evidence-based interventions. Along with seminal discoveries and scientific inventions, this has led to improved patient safety, reduced infection rates, and enhanced visualization of anatomical structures, setting the stage for modern surgical practices.

Despite these achievements, otologic surgery is far from a finished chapter. Advances in endoscopy, robotics, and regenerative medicine keep pushing the boundaries of what is possible, promising not only less invasive procedures, but also faster recovery times and better functional outcomes. The future of the field may see the integration of bioengineered implants, gene therapy, and AI-driven surgical planning, further revolutionizing treatment strategies.
